# First Seroprevalence Survey of Avian Reovirus in Broiler Breeders Chicken Flocks in Morocco

**DOI:** 10.3390/v15061318

**Published:** 2023-06-02

**Authors:** Ahmed Achhal Elkadmiri, Amal Zhari, Noura Aitlaydi, Mohammed Bouslikhane, Asma Fagrach, Mohamed Mouahid, Siham Fellahi

**Affiliations:** 1 Department of Pathology and Veterinary Public Health, Agronomy and Veterinary Institute Hassan II, Rabat 10000, Morocco; achhal.cvrc@gmail.com (A.A.); amalzhari1999@gmail.com (A.Z.); bouslikhanemed@yahoo.fr (M.B.); fagrachasma@gmail.com (A.F.); 2 Cabinet Tit Mellil, Tit Mellil 29640, Morocco; aitlaydinoura@gmail.com; 3 Cabinet Mouahid, Temara 12000, Morocco; mohamedmouahid@gmail.com

**Keywords:** avian reovirus, seroprevalence, broilers, breeders, Morocco, ELISA

## Abstract

Avian reovirus (ARV) is a prevalent infectious agent that has the potential to cause respiratory and gastrointestinal illnesses in poultry, leading to substantial financial losses in the poultry sector. Until now, there have been no investigations conducted to examine the epidemiological status of ARV infections in Morocco. The aim of this study was to investigate the seroprevalence of ARV infections with respect to area, types of chickens (broiler breeder, and broiler), vaccination status, and age of chickens. A total of 826 serum samples were collected from 36 broiler and broiler breeder flocks, with 14 of them being unvaccinated, fromsix different regions of Morocco, namely Casablanca-Settat, Rabat-Salé-Kénitra, Tanger-Tétouan-Al Hoceïma, Oriental, Marrakech-Safi, and Fez-Meknès between 2021 and 2022.These serum samples were screened using a commercial indirect ELISA ARV antibody test kit (IDEXX REO). The study found that all tested flocks were positive for ARV-specific antibodies, indicating that the virus was present in these flocks. Out of the 826 serum samples tested, 782 were positive for ARV-specific antibodies. The overall prevalence of ARV infections in breeder and broiler flocks was calculated to be 94.6% ± 0.78. To summarize, the current study provides evidence of the widespread distribution of ARV infections in Morocco, suggesting that the poultry industry in the country is highly infected with ARV.

## 1. Introduction

Avian reovirus is a virus that belongs to the Reoviridae family and the Orthoreovirus genus. It can infect various species of birds, including chickens, turkeys, ducks, and quails. The virus can cause several diseases in birds, including viral arthritis/tenosynovitis, respiratory tract infections, and malabsorption syndrome [1,2,3]. Avian reovirus is transmitted from bird to bird through contact with infected feces, respiratory secretions, or contaminated surfaces [1,4]. The virus can also be spread vertically from infected hens to their offspring through the egg [5]. The diagnosis of avian reovirus infection typically involves a combination of clinical signs, gross and histopathological lesions, and laboratory tests, including virus isolation, serology, and molecular methods [1,6]. It is important to use a combination of these techniques for an accurate diagnosis of avian reovirus infection [6,7,8,9]. The early detection and diagnosis of the virus can help in implementing appropriate control measures, including vaccination and biosecurity practices, to prevent the spread of the virus and minimize its impact on poultry production. There are currently no specific treatments for avian reovirus infections, and prevention mainly relies on good management practices, including maintaining proper biosecurity measures, vaccination, and maintaining optimal environmental conditions for birds. Vaccination is an effective method of controlling avian reovirus infections in poultry. There are several types of vaccines available for avian reovirus, including live attenuated vaccines, inactivated vaccines, and subunit vaccines [10].

The seroprevalence of avian reovirus varies depending on several factors, including the age and breed of the birds, management practices, and geographical location. In general, seroprevalence is higher in older birds than in younger birds, as older birds have had more opportunities for exposure to the virus [7,8]. Seroprevalence studies are usually conducted using enzyme-linked immunosorbent assay (ELISA) or serum neutralization tests to detect antibodies against avian reovirus in bird blood samples [9,10]. The results of these tests can provide important information about the prevalence of the virus in a population and help to guide disease control strategies. It is important to note that seroprevalence does not necessarily indicate the presence or absence of disease, as birds can have antibodies against the virus without showing any clinical signs. Therefore, a combination of seroprevalence data and clinical observations is needed to fully assess the impact of avian reovirus infections on a bird population [10].

In Morocco, avian reovirus presents a significant concern for the poultry industry, and ongoing research is necessary to develop new control strategies and to improve our understanding of the virus and its impact on bird health and production. This study, for the first time, investigates the seroprevalence of Reovirus in broiler breeder and broiler chickens in different regions in Morocco.

## 2. Materials and Methods

### 2.1. Sampling Protocol

In this study, a total of 826 sera were collected from 36 different broiler breeder and broiler flocks in 6 different regions of Morocco (Casablanca-Settat, Oriental, Tanger-Tétouan-Al Hoceïma, Rabat-Salé-Kénitra, Marrakech-Safi, Fès-Meknès). In total, 379 serums were collected in 2021 and 447 in 2022, according to the distribution of collection dates. Several parameters, such as age, type of bird (broiler breeder, broiler), and vaccination status were considered during the sample collection (Table 1). The age group distribution of the birds from whom the 826 serums were collected was as follows: 1 day old for broilers and 1 day old, 8 to 12 weeks, and 21 to 26 weeks for broiler breeders’ flocks.

The stratified random sampling strategy used in this study provided statistical representativity with a confidence level of 95% and a diagnostic test sensitivity of 86%. For the 6 Moroccan regions included in the study, the criteria “total poultry farms for each region” helped to divide the flocks into 3 subgroups called strata, each of which represented a density (high, medium, and low density). For a given theoretical seropositivity rate of 50%, and according to the size of flocks in each region (approximately 10,000), the minimum required sample size is determined to be 6 per flock for each age group but can be increased to 12 to maximize the chances of having reliable results. For each bird, a sample random selection is applied to ensure an equal chance of being selected. Nevertheless, in some circumstances, the sample size was reduced due to potential losses during collection or storage.

### 2.2. Collection of Blood, Transportation, and Serum Preparation

Blood samples were obtained from the wing vein, using a 3 mL syringe, of the randomly selected apparently healthy birds. The blood was transferred into sterile tubes and kept at room temperature on a slanted surface, which allowed the blood to clot, and the resultant supernatant designed serum was harvested by centrifugation at 12,000× *g* for 5 min and then transferred into a clean Eppendorf tube. Each sample was labeled with an appropriate alphanumeric code. The collected sera were stored at −20 °C in preparation for performing the ELISA.

### 2.3. Serology

In order to detect the ARV antibodies, the collected and processed sera samples were analyzed using a commercially available ARV-specific indirect ELISA kit (IDEXX Laboratories, Westbrook, ME, USA) containing ARV antigen-coated plates, in accordance with the instructions supplied by the manufacturer.

In brief, 100 µL of serially pre-diluted test samples (1:500) was dispensed into the 96-well microtiter plates coated with ARV antigens, and previously individually filled with the positive (PC) and negative controls (NC). The microplates were subsequently incubated for 30 min in order to form an antibody–antigen complex. After removing the solution content (unbound materials) from the wells, the microplates were washed 5 times with 350 µL of distilled water, then 100 µL of horseradish peroxidase conjugate (GOAT anti-chicken) was dispensed into each well, which binds to any attached chicken antibody, and incubated for 30 min and then washed as previously described. Thereafter, 100 µL of TMB (3,3’,5,5’-Tetramethylbenzidine) substrate was incubated in each well for 15 min, which allowed the color to develop at room temperature. Finally, the reaction in the wells was terminated by dispensing 100 µL of a blocking solution. An ELISA microtiter plate photometer reader, equipped with a 650 nm filter, was used to measure, and record the absorbance values and the optical density of each well. 

### 2.4. Statistical Analysis

In order to investigate the seroprevalence of birds against various diseases, several parameters were considered including age, type of birds, regions and their geographic location, vaccination status, period of collection, and flock density. The seropositivity of indirect ELISA antibody titers, as well as their means and standard deviations, were calculated using a one-way analysis of variance (ANOVA) model. Additionally, ANOVA and post hoc t-tests were used to determine whether there were any significant differences in antibody titers within and between the different parameters investigated. Differences were considered statistically significant when their probability (P) values were equal to or less than 0.05. By utilizing these statistical techniques, it was possible to identify the factors that influence seroprevalence, and to determine the effectiveness of vaccination programs against avian reovirus in different regions and age groups of birds.

### 2.5. Interpretation of ELISA Results

For each serum tested, the following formula (OD value of sample—OD of NC)/(OD of PC—OD of NC) was used to calculate the simple to positive S/P ratio, and by converting the resultant S/P ratio, this equation Log_10_ Titer = 1.09 (log_10_ S/P) + 3.36 helped to measure the endpoint titers. Samples with titers greater than 396 (S/P ratio > 0.2) are considered positive.

## 3. Results

### 3.1. Serological Detection of ARV Specific Antibodies 

The study evaluated a total of 826 serum specimens collected from different regions of Morocco to determine the prevalence of avian reovirus-specific antibodies. The results showed that 782/826 of the specimens were positive for ARV-specific antibodies, indicating a high prevalence of the virus in the sampled regions.

### 3.2. Geographical Location Effect

A further analysis of the prevalence rates in different regions revealed that all regions had confirmed ARV-specific antibodies, with the highest rates being found in the Marrakech-Safi 265 (98.51%) and Rabat-Salé-Kénitra 78 (100%) regions. The prevalence rates were lower in the Casablanca-Settat and Oriental regions, with rates of 88.5% and 90.82%, respectively. Interestingly, when considering Tanger-Tétouan-Al Hoceïma and Fez-Meknès in the north, Casablanca-Settat and Rabat-Salé-Kénitra in the west, Marrakech-Safi in the south, and Oriental in the east, the prevalence rates were significantly higher in the north and south regions of Morocco, with rates of 96.77% and 98.51%, respectively, compared to the east (90.82%) and west regions (91.45%).

### 3.3. ARV Seroconversion and Vaccination Effect

The study also evaluated the seropositivity rates in vaccinated and unvaccinated flocks, and found that the unvaccinated flocks had a higher seropositivity rate of 98% compared to 92.8% in vaccinated flocks. Furthermore, the study compared the prevalence of ARV-specific antibodies between breeding and broiler chickens and found no significant difference (*p*-value = 0.63) between the two groups. Moreover, the results showed that the highest antibody titer means were recorded in the vaccinated flocks, particularly in Tanger-Tétouan-Al Hoceïma, with an average of 6078.62 ± 240.2. On the other hand, the unvaccinated flocks from Fez-Meknès had the lowest antibody titer mean with a rate of 3838.8 ± 322.9. Interestingly, when the geographic location of these regions was taken into account, the average antibody titer means of 5037.10 ± 301.25 did not exhibit any significant differences.

### 3.4. Chronology Effect

Furthermore, the study evaluated the prevalence rates of ARV-specific antibodies over sample collection periods and found that the prevalence was significantly higher in 2021 than in 2022. Among the 782 serum specimens that tested positive for avian reovirus, there was a wide range of antibody titers, ranging from 403 to 22,372, with an average of 5320.84. Moreover, the study found that the level of ARV antibody was highly variable among individuals, as evidenced by 680 out of the 782 seropositive serum samples having titers between 396 and 9999. Subsequently, the period of collection had a significant effect on the antibody titers, with a higher mean titer in 2022 (5966.07) compared to 2021 (3956.05). 

### 3.5. Density and Age Effects

The findings indicate that flock density has a significant impact on antibody titers, with the medium density flocks showing the highest mean titer of 6135.27 ± 200, followed by low-density flocks with a mean titer of 5000.08 ± 317.72, and high-density flocks with a mean titer of 2879.42 ± 154.1. The difference between the mean titers of medium-density and low-density flocks is statistically significant (*p* < 0.05), while the difference between medium-density and high-density flocks is not significant.

The type of production, either breeders or broilers, did not demonstrate a significant effect (*p*-value = 0.22) on the antibody titers, with means of 4975.55 ± 151.4 and 5470.03 ± 345.8, respectively. Regarding the age of chickens, the results indicate that the mean antibody titer varies significantly depending on the age of the chicken. Chickens aged between 21 and 26 weeks had the highest mean titer of 5853.26 ± 266.9, followed by chickens aged 1 day (5325.41 ± 301.6), and those aged between 8and12 weeks (3766.13 ± 219.1).

### 3.6. Statistical Analyses

**Figure 1:**
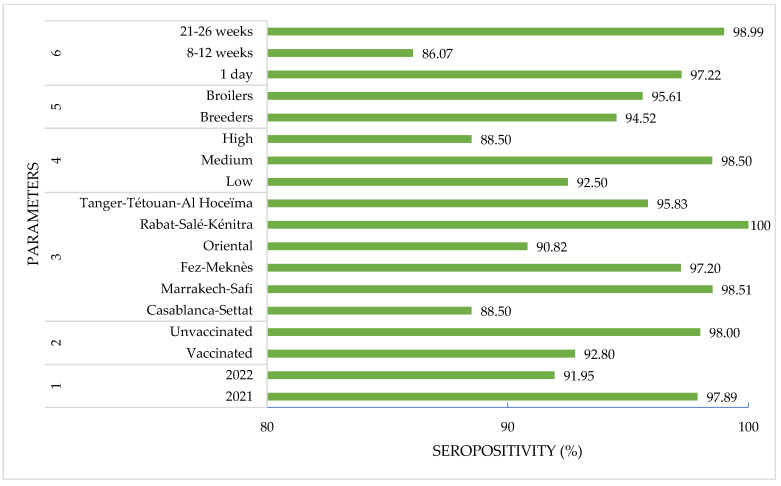
Seropositivity percentage according to investigated parameters.

Table 1 provides a summary of the statistical analysis results on various parameters that may affect antibody titers in chickens. The correlation coefficients between different parameters and the test results (positive or negative) for this certain study are shown in Table 2. Among the criteria, the vaccination status of the birds had a statistically significant correlation (*p* < 0.01) with the test results, with a Pearson correlation coefficient of 0.104 (Figure 1). Another significant factor was the geographic region, with a negative correlation coefficient of −0.146 (*p* < 0.001). Interestingly, there was a weak positive correlation (*p* < 0.05) between the age of the birds and the test results, with a Pearson coefficient of 0.017. On the other hand, there was no significant correlation between the density of the flock, the type of production, or the period of collection and the test results (Figure 1).

## 4. Discussion

Avian reovirus is a viral pathogen that has been frequently detected in chickens worldwide, causing a range of clinical symptoms, including stunted growth, enteritis, respiratory symptoms, pericarditis, and myocarditis. ARV infections in poultry can lead to significant economic losses in the poultry industry, resulting in reduced productivity and increased mortality. Despite the global prevalence of ARV infection in poultry, there is a lack of information on its prevalence and impact on poultry farms in Morocco.

The proposed study is of great importance as it will be the first to investigate the prevalence, transmission, and impact of ARV infections on poultry production in Morocco. The study involved collecting and analyzing samples from poultry farms across different regions of Morocco to determine the prevalence of ARV infections. Additionally, the study assessed the transmission dynamics of ARV infections and investigated the potential risk factors associated with its spread in poultry farms.

In accordance with previous research with similar objectives [10,11,12,13,14], the IDEXX REO^®^ kit was employed as the sole method for serology testing in the current study, due to its widespread use in other countries. Although the ELISA has been shown to be a more sensitive, faster, and more cost-effective method than the existing antibody assay technique in the viral neutralization of AGP, it has certain limitations [15]. Furthermore, according to the manufacturer of the ARV ELISA IDEXX kit, the vaccines used for ARV in Morocco are all detected by this kit, namely Nobilis ReoInac (MSD), Nobilis Reo-S(MSD) and Poulvac Tri-Reo (Zoetis); the first one is composed based on two ARV strains: 1733 and 2408, the second one contains the 1133 ARV strain, and the third contains 1133, 2408, and 3005 strains (the whole virus were used for these vaccines).

For instance, the ELISA method cannot detect all Reovirus strains and serotypes, which may result in false-negative results. Therefore, it is important to consider the limitations of this technique when interpreting the results of the current study. It should also be noted that negative results obtained using a commercial ELISA kit cannot completely rule out the presence of anti-Reovirus antibodies [15].

The primary objective of this study, conducted in 2021 and 2022, was to investigate the prevalence of ARV infections in breeder and broiler flocks in six different regions of Morocco (Casablanca-Settat, Rabat-Salé-Kénitra, Tanger-Tétouan-Al Hoceïma, Marrakech-Safi, Fez-Meknès, Oriental). A total of 826 serum samples were collected from 36 flocks for serological testing, out of which 14 flocks were unvaccinated against ARV, while the remaining flocks had received ARV vaccination. An indirect ELISA commercial kit was used for the testing of serum samples. The results revealed that 782 serum samples were positive for ARV-specific antibodies, indicating a high prevalence (94.6% ± 0.7) of ARV infections in breeder and broiler flocks across all regions during 2021 and 2022. The high prevalence of ARV infections in all the examined flocks suggests that ARV infections are common in poultry farms in Morocco. In the 782 serum specimens found to be positive, antibody titers ranged from 403 to 22,372, with the average being 5320.8. 

Based on the vaccination status, the prevalence of ARV infection was higher in non-vaccinated chickens (98%) compared to vaccinated chickens (92.8%), indicating that the seroprevalence rate is related to the effectiveness of vaccination against ARV in different flocks, which can be explained by the involvement of multiple factors. These factors include the effect of the surrounding environment, the quality of vaccination, which depends on the techniques used to administer the vaccine, such as the equipment used and the skill of the person administering it, and the vaccination program itself, which includes the type of vaccine used, the age at which prophylaxis is administered, and the method of vaccination. According to several studies, the success of vaccination is influenced by various factors, including careful storage conditions (maintaining temperature at +2 °C or +8 °C, protected from light for up to 2 years, and prompt use after reconstitution as per instructions), the appropriate dosage and administration route, vaccination timing (administering the primary vaccine between the 7th and 10th week for broiler breeders, and a booster vaccine before the onset of lay to optimize the passive immunization of chicks), vaccination coverage and compliance (ensuring complete coverage to minimize gaps in the vaccination process and protect all individuals), and considering the potential immunosuppressive effects (avoiding the concurrent use of the S1133 vaccine strain with live Marek and Gumboro vaccines). Failure to adhere to each of these parameters can lead to a lower seropositivity rate, even if the flock is considered vaccinated [16,17,18]; additionally, it was observed that all samples of unvaccinated 1-day-old broilers tested positive for ARV infection, with higher mean antibody titers (7458.7 ± 666.9) than unvaccinated broiler breeders (5149.2 ± 229.1). This could be attributed to the fact that the immune system of young chickens is not fully developed, and they are more susceptible to infections. Furthermore, protection against reovirus involves the maternal transfer of antibody through the yolk to the progeny; this means that the breeders are either vaccinated or have had contact with the wild ARV during their laying period [19]. On the other hand, older chickens have developed age-related resistance to the virus, which could explain the lower antibody titers observed in the unvaccinated broiler breeders. 

The geographical location effect was investigated, and the results indicated that the prevalence of reovirus was significantly higher in medium-density flocks as compared to low-density and high-density flocks. Specifically, 98.5% of the sampled birds from medium-density flocks tested positive for the virus, whereas only 92.5% and 88.5% of the sampled birds from low-density and high-density flocks, respectively, were positive for reovirus. The increased prevalence of the virus in medium-density flocks, particularly among the non-vaccinated flocks, could be attributed to the higher likelihood of virus transmission and infection in such flocks. This is due to the close proximity of birds in medium-density flocks, which can facilitate the spread of the virus through contact, airborne particles, or contaminated materials.

The seroprevalence of ARV infection was not significantly different among the different regions studied. The highest seroprevalence was observed in Rabat-Salé-Kénitra, where all of the sampled flocks were positive for ARV antibodies. In contrast, the lowest seroprevalence was observed in Casablanca-Settat, with only 88.5% of the sampled flocks being positive for ARV antibodies. For the other regions, the seroprevalence ranged from 90% to 97%, with 98.5% for Marrakech-Safi, 97.2% for Fez-Meknès, 95.8% for Tanger-Tétouan-Al Hoceïma, and 90.8% for Oriental. This indicates that the virus seems to have a relatively equal presence across all regions, indicating that geographical location does not have a significant effect on the spread and transmission of the virus. 

Regarding periods of collection, the prevalence of ARV specific antibodies was significantly higher in 2021 than in 2022, being, respectively, 97.9% and 91.9%. The higher seroprevalence observed in 2021 compared to 2022 can be explained by two factors: the high viral pressure in Moroccan farms due to the importation of day-old breeder chicks of broiler chickens that were likely already infected with the avian reovirus, and the shortage of vaccines. The appearance of lameness cases on the Moroccan territory in 2021 was initially thought to be due to avian reovirus infection, but the PCR results turned out to be negative. Further investigations revealed that the problem was caused by nutritional and genetic issues. These findings highlight the need for better biosecurity measures in the importation of day-old chicks, and the timely provision of sufficient vaccine stocks to prevent the spread of avian reovirus in Moroccan poultry farms [14].

The serological testing results revealed a high seroprevalence of ARV in unvaccinated broiler and breeder flocks in Morocco, with values of 100% and 97.4%, respectively, indicating the endemic and circulating nature of this virus within breeder farms, independent of various factors. The high incidence of ARV infections in breeders is probably due to two main sources. Firstly, the flocks may become infected by the virus through the environment, as ARVs are commonly found in the poultry population and can be isolated from the respiratory and gastrointestinal tracts of chickens showing acute or asymptomatic infections [12]. Secondly, contaminated vaccines, especially live-virus vaccines, may represent another potential source of ARV infections, as there have been reports of occasional avian reovirus contamination in avian viral vaccines, as reported by Juan Pu, Xingli et al. in their article [12]. 

To better understand the circulation of ARV infection in Moroccan poultry farms, we examined the prevalence of ARV antibodies in unvaccinated broiler breeders of different age groups. The highest prevalence of ARV antibodies was found in 1-day-old chicks, with a prevalence of 100%. This suggests that younger chickens have lower immunity due to their underdeveloped immune system [19]. Chickens in the age group of 21–26 weeks had the next highest prevalence rate of 99%, followed by chickens in the age group of 8–12 weeks with a prevalence rate of 93.7%. Additionally, the mean antibody titers were highest at the age of 1 day, at 6288.5 ± 292.7, decreased to 2984.5 ± 230.6at the age of 8–12 weeks, and then increased again to 8175.4 ± 341.1. This trend may be explained by prolonged exposure and recurrent infections. 

The vaccinated breeder flocks displayed a similar pattern to the unvaccinated flocks, with a high seroprevalence rate of 98.1% at 1 day of age. This can be attributed to the vaccination of the parent birds before the chicks are imported from foreign countries. The seroprevalence rate then decreased to 81.1% at 8–12 weeks of age, which can be explained by the vaccination schedule that might not have been enough to sustain high antibody levels at this age. The increase in seroprevalence to 98.9% for the age group of 21–26 weeks can be explained by the close contact between birds or the transfer of birds to production facilities, which is a common practice at this stage, and may increase their exposure to environmental stressors suppressing the immune system, enhancing the exposure to the virus. 

The prevalence of ARV infection in the poultry population has been investigated in several countries. Comparable findings were reported in Romania, by Botuş et al. [20], Iran, by Bokaei et al. [21], and Canada, by Nham et al. [11], where the seroprevalence rates were 99.5%, 98.3%, and 90.5%, respectively. However, lower seroprevalence rates were recorded in Turkey (75.9%), by Erol et Şengül [22], Vietnam (85.2%), by Thu H. T. V. [13], and Egypt (80.6%), by Al-Ebshahy et al. [10]. In contrast, some countries, such as Nigeria [23] and Bangladesh [24], reported relatively lower prevalence rates of ARV infection in broilers or breeders with 41% and 39.5%, respectively. In India, a seroprevalence rate of 8.7% [14] was reported in unvaccinated broiler breeder flocks. These findings indicate that ARV infection is widely prevalent in the poultry population worldwide, with varying degrees of prevalence across different countries. It underscores the need for continuous monitoring and the implementation of effective control measures to mitigate the impact of ARV infection on poultry production.

The variations in the observed seroprevalence rates across different countries suggest the potential influence of multiple factors, including differences in management practices, vaccination schedules, environmental conditions, and potential exposure to other viral and bacterial infections [14]. Additionally, the age of the flock at the time of transport, immune exposure, and the level of viral excretion may also contribute to these variations. Therefore, further research is needed to identify the contributing factors and develop effective control strategies to prevent the spread of ARV globally. The need for such control measures is especially crucial in countries where high prevalence rates have been reported, such as Morocco. The development of effective control strategies and a better understanding of the factors influencing ARV spread would significantly contribute to enhancing overall poultry health and productivity.

The epidemiology of ARV infections in North Africa is not well understood, particularly in Algeria, Mauritania, and Tunisia, which have shared a molecular study of ARV [25] but not a serology study. The lack of information on the presence and prevalence of ARV in these countries makes it difficult to determine the extent of the disease in the region. Since Morocco has a high incidence of ARV infections, it is crucial to conduct similar studies in neighboring countries to gain a more comprehensive understanding of the regional context of ARV in North Africa.

Although our study on reovirus seroprevalence in poultry shares similarities with other research, there are notable differences in the study design and population that complicate comparisons. These differences include the criteria for defining flock status as positive or negative, the antibody titer thresholds used to indicate reovirus seropositivity, the age of broilers and breeders during sampling, and the vaccination status of breeders [11]. These dissimilarities may have influenced the results and should be considered when interpreting findings from various studies. For instance, the threshold for classifying a flock as seropositive may differ depending on the ELISA kit used or the sensitivity of the test. Additionally, the age of broilers during sampling can affect seroprevalence rates, with older birds potentially having higher antibody titers due to prior exposure and close contact [11]. Vaccination status can also impact seroprevalence rates, with vaccinated flocks potentially having a high seroprevalence rate due to their prior immunization. 

## 5. Conclusions

The findings of this study provided valuable insights into the control and management of ARV infections in poultry farms in Morocco. The results served as a foundation for future research aimed at comparing the efficacy of different vaccination strategies in controlling ARV infections in poultry farms across the country; focusing on evaluating the long-term effectiveness of different ARV vaccines, and identifying the most suitable vaccination schedules for different age groups and breeds of poultry; and investigating the potential benefits of combining vaccination with other control measures, such as biosecurity protocols, to enhance the efficacy of ARV control programs. This study will also contribute to filling the current knowledge gaps regarding ARV infections in chickens in Morocco, supporting the sustainable development of the poultry industry in the country.

## Figures and Tables

**Table 1:** Serological detection of ARV specific antibodies according to all parameters.

Parameters	Criteria	Sera	+	Sp (%) *	Mat ± sd
Density	High	226	200	88.5%	2879.42 ± 154.1 ^c^
	Medium	454	447	98.5%	6135.27 ± 200 ^a^
	Low	146	135	92.5%	5000.08 ± 317.7 ^b^
Vaccination status	Vaccinated	519	482	92.8%	5340.60 ± 168
				[91–95] ^b^	
	Unvaccinated	307	300	98%	4542.05 ± 241
				[96–99] ^a^	
Type of birds	Breeders	712	673	94.52% ^ns^	4975.55 ± 151.4 ^ns^
				[92.85–96.2]	
	Broilers	114	109	95.61% ^ns^	5470.03 ± 345.8 ^ns^
				[91.8–99.43]	
Age groups	Breeders 1 day	172	170	98.84% ^a^	5180.78 ± 257.4 ^a^
				[97.22–100]	
	Breeders 8–12 weeks	244	210	86.07% ^b^	3766.13 ± 219.1 ^b^
				[81.69–90.44]	
	Breeders 21–26 weeks	296	293	98.99% ^a^	5853.26 ± 266.9 ^a^
				[97.84–100]	
	Broilers 1 day	114	109	95.61% ^a^	5470.03 ± 345.8 ^b^
				[91.8–99.43]	
Period of collection	2021	379	371	97.89% ^a^	3956.05 ± 161.8 ^b^
				[96.44–99.34]	
	2022	447	411	91.95% ^b^	5966.07 ± 207.6 ^a^
				[89.41–94.48]	
Regions	Casablanca-Settat	226	200	88.50% ^c^	5136.41 ± 240.2 ^a^
				[84.3–92.69]	
	Fez-Meknès	107	104	97.20% ^ab^	3838.80 ± 322.9 ^b^
				[94.02–100]	
	Marrakech-Safi	269	265	98.51% ^a^	4977.81 ± 265 ^a^
				[97.06–99.97]	
	Oriental	98	89	90.82% ^bc^	5385.33 ± 432.4 ^a^
				[85–96.64]	
	Rabat-Salé-Kénitra	78	78	100% ^a^	5590.12 ± 455.8 ^a^
	Tanger-Tétouan-Al Hoceïma	48	46	95.83% ^ab^	6078.62 ± 240.2 ^a^
				[85–96.64]	
Geographic location	East	98	89	90.82% ^b^	5385.34 ± 432.4
				[85–96.64]	
	North	155	150	96.77% ^a^	4532.43 ± 294.2
				[93.96–99.59]	
	South	269	265	98.51% ^a^	4977.81 ± 265
				[97.06–99.97]	
	West	304	278	91.45% ^b^	5252.83 ± 213.4
				[88.29–94.61]	

Sera: Total number of sera samples analyzed; +: positive sera; SP (%): seroprevalence percentage; MAT ± SD: Mean antibody titer ± standard deviation; * Seroprevalence values are shown along with 95% confidence intervals. Different superscripts letters (^a^, ^b^, ^c^) in the same column indicate a significant difference (*p* < 0.05) between criteria of each parameter; ^ns^: non-significant.

**Table 2:** Correlation coefficient (r) with different parameters.

		Density	Vaccination Status	Age	Regions	Period of Collection	Type of Birds
Results	Pearson correlation coefficient	−0.010 NS	0.104 **	−0.146 **	0.114 **	−0.132 **	0.017 NS
	Sig. (bilateral)	0.769	0.003	0.000	0.001	0.000	0.630
	N	826	826	826	826	826	826

Sig.: significance; NS: non-significant; **: highly significantly different at *p* < 0.01.

## Data Availability

Not applicable.
